# Influence of site and smoking on malignant transformation in the oral cavity: Is the microbiome the missing link?

**DOI:** 10.3389/froh.2023.1166037

**Published:** 2023-03-23

**Authors:** Sheila Galvin, Gary P. Moran, Claire M. Healy

**Affiliations:** ^1^ Division of Oral and Maxillofacial Surgery, Oral Medicine and Oral Pathology, School of Dental Science, Trinity College Dublin, Dublin Dental University Hospital, Dublin, Ireland; ^2^ Division of Oral Biosciences, School of Dental Science, Trinity College Dublin, Dublin Dental University Hospital, Dublin, Ireland

**Keywords:** oral cancer, oral microbiome, OSCC, smoking, oral leukoplakia, oral potentially malignant disorders

## Abstract

The tongue and floor of the mouth are high-risk sites for oral squamous cell carcinoma (OSCC), while smoking is its most significant risk factor. Recently, questions have been raised as to the role of the oral microbiome in OSCC because of a wealth of evidence demonstrating that the microbiome of OSCC differs from that of healthy mucosa. However, oral site and smoking also have a significant impact on oral microbial communities, and to date, the role these factors play in influencing the dysbiotic microbial communities of OSCC and precursor lesions has not been considered. This review aims to examine the influence of site and smoking on the oral microbiome and, in turn, whether these microbiome changes could be involved in oral carcinogenesis.

## 1. Introduction: micro-organisms and cancer

Micro-organisms are estimated to cause up to 20% of fatal human malignancies, with approximately 2.2 million infection-attributed cancers diagnosed worldwide in 2018 (1). To date, 11 microbes have been designated as human carcinogens by the International Agency for Cancer Research (IARC): *Helicobacter pylori*, Hepatitis B and C viruses, *Opisthorchis viverrini*, *Clonorchis sinensis*, Human papillomavirus, Epstein–Barr virus, Human herpes virus 8, Human T-cell Lymphotropic Virus 1, *Schistosoma haematobium*, and Human Immunodeficiency Virus-1 (2). Recent advances in microbiome research, however, have demonstrated that many other microbial species are likely to play a role in carcinogenesis, including the oral bacterial pathogen *Fusobacterium nucleatum*, which is increasingly being linked with colorectal carcinoma (3).

There is general agreement that the microbiome of oral squamous cell carcinoma (OSCC) differs from that of healthy tissue, and there is emerging evidence that pre-cancerous lesions also harbour different microbial communities to healthy mucosa (4–10). There is also a consensus that the mucosal site and tobacco use have a significant impact on the composition of the oral microbiome (11–17). However, what has not been considered to date is the role these factors play in influencing the dysbiotic microbial communities of OSCC and precursor lesions and whether these microbiome changes are involved in malignant progression. This mini review will, therefore, examine the influence of site and smoking on the oral microbiome and, in turn, the evidence for any related dysbiosis in oral carcinogenesis.

## 2. Risk factors of OSCC

Established risk factors for OSCC are tobacco and alcohol consumption, betel nut use, age and presence of an oral potentially malignant disorder (OPMD), a group of mucosal conditions associated with a statistically increased risk of transformation to cancer. Tobacco is the most significant of these risk factors, with up to 25% of oral cancers directly attributable to smoking, the risk of OSCC increasing with the number of cigarettes smoked, and up to 80% of OSCCs occurring in smokers (18, 19). Tobacco and alcohol are known to act synergistically, increasing the risk of oral cancer up to 15-fold, particularly in the floor of the mouth (18, 20). However, up to 15% of OSCCs in older adults and up to 26% of OSCCs in younger adults have little to no exposure to tobacco or alcohol (21, 22), with Deneuve et al. also finding that their non-smoking tongue cancer cohort had a significantly higher prevalence of oral leukoplakia (OLK) than their smoking cohort (21). OLK is the most common OPMD with a global estimated incidence of 4.1% and malignant transformation rate of 9.8% (23). Risk factors for the development of OLK include the same risk factors as oral cancer, namely tobacco smoking, consumption of alcohol, and use of betel nut. However, OLKs in non-smokers are generally considered to be at a higher risk of malignant transformation than OLKs in smokers (24, 25).

Finally, epidemiological studies and meta-analyses have associated poor oral hygiene, tooth loss, and periodontal disease with OSCC (26–30). However, it is still unclear as to whether these associations are due to chronic periodontal disease sharing risk factors with OSCC, namely smoking and alcohol use, or to the microbial dysbiosis and/or inflammation associated with these conditions.

## 3. Microbiome of OSCC and OPMD

There is a wealth of literature confirming that the microbiome of OSCC is different from that of healthy mucosa. However, the literature differs as to what microbial changes are responsible for these differences, the conflicting evidence being due at least in part to the various methods by which the oral microbiome is sampled, including mucosal swab, tissue biopsy, and saliva collection. Mucosal surface swabs are non-invasive and enable direct and targeted sampling of specific oral mucosal surfaces. Their principal advantage, however, is that they allow for separate sampling of lesional and matched normal sites within the same individual and therefore control of confounding factors such as smoking, alcohol consumption, and oral hygiene. Direct tissue sampling enables the investigation of invasive species within oral lesions. This method of sampling is, however, invasive, requiring mucosal biopsy, and so carries risks for patients such as post-operative pain, bleeding, and infection. Therefore, in recent studies, ethical considerations have prevented the biopsy sampling of normal tissue as a control. Nonetheless, tissue microbiome studies of oral cancers have shown interesting results, particularly in relation to *Fusobacterium nucleatum.* Finally, the ease of collection of saliva makes it a popular choice for microbiome studies. However, saliva collection does not allow for sampling of specific sites/lesions and has also been shown to be strongly biased towards tongue and palate communities (12, 13, 31). The saliva sampled can also be contaminated with food and other debris, and although there are various protocols described for pre-sampling rinsing to minimise contamination, they also provide for different sampling methods among studies.

Studies have also used different methods, which have evolved significantly over the years, to characterise the oral microbiome, from initial culture-based methods to DNA-DNA hybridisation and more recently to next-generation sequencing (NGS)-based methods such as marker gene sequencing and metagenomics. Marker gene sequencing usually involves sequencing specific variable (V) regions of the highly conserved bacterial 16S rRNA gene to identify bacteria present in a sample, whereas metagenomic methods sequence the entire genome and therefore can provide not only taxonomy but also the metabolic pathways and functional profiles of the microbial community sampled (32, 33). Metagenomic methods are currently limited by cost and accessibility, but due to the kind of information they can provide, it is likely that they will supersede marker gene sequencing in time. It should be noted that the choice of the variable region (V1–V9) to sequence also needs consideration. Regions V1–V5 are typically used for the oral microbiota, with recent reports suggesting that the V1–V3 region is preferable to the more commonly used V3–V4 region as it is more divergent and therefore provides more phylogenetic resolution and a more accurate assessment of population diversity (34, 35). Several platforms are available for sequencing, of which Illumina remains the most commonly used due to its wide availability, high output, and high level of accuracy (33).

The majority of the microbiome studies on OSCC and OLK to date have sequenced various parts of the V1–V5 region of the 16S rRNA gene using NGS. While the choice of region can impact phylogenetic resolution and diversity as mentioned previously, the choice of the sampling method has an even greater impact on results. Studies using swab and biopsy sampling methods have found an increased abundance of *Fusobacterium* and reduced abundance of *Streptococcus* and *Rothia* at OSCC sites (4, 5, 36–38), while some of those sampling saliva have identified Firmicutes, *Streptococci,* and *Rothia* as more abundant in OSCC patients (7, 39, 40). However, no OSCC microbiome studies to date have controlled for significant influences such as smoking, oral site, or oral hygiene, all factors known to influence the oral microbiome and that need to be considered before any definite conclusions can be drawn on the influence of the microbiome on the development of OSCC.

To date, only two authors have looked at the microbiome of OPMDs sampled by swabbing. Schmidt et al. (4) demonstrated that abundances of Firmicutes, Actinobacteria, and *Streptococcus* spp. were significantly reduced at OPMD sites relative to contralateral normal sites, while Amer et al. (10) found a significant enrichment of OLKs with *Fusobacterium, Leptotrichia, Rothia mucilaginosa,* and *Campylobacter* spp. relative to contralateral normal sites. However, Amer et al*.* also demonstrated that site and smoking status had a more significant influence on the microbiome than the presence of an OLK. Changes in the abundance of *Fusobacterium nucleatum* subsp. *vincentii* were particularly site-dependent with increased abundance found on buccal OLKs and on lateral tongue contralateral normal sites. Lateral tongue normal sites also showed an enrichment of *Rothia mucilaginosa*. These data highlight the necessity for control samples to be matched by site, which is possible only by mucosal swab sampling.

## 4. Influence of site on the microbiome

The tongue is the most common intra-oral site for cancer, accounting for approximately 46.7% of oral cavity (excluding salivary gland) cancers in the United States in 2016 (41). Despite an overall decrease in tobacco and alcohol–associated OSCC in the US between 1985 and 2009, the proportion of tongue cancers in adults under 50 years of age increased over the same 25-year period (42, 43).

Unsurprisingly, the tongue is also the most common, or second most common, site of OLK and is considered a high-risk site for malignant transformation (23, 44, 45), with the most recent systematic review on the topic finding that 56.3% of OLKs that transformed to cancer were on the tongue (23). A slightly older systematic review identified the buccal mucosa (18.4%) and the tongue (16.4%) as the most common sites for OLK (45), with the authors commenting that the site involved was related to the lifestyle factors of populations studied; the buccal mucosa is more likely to be involved in regions where betel nut is used, whereas the tongue and floor of mouth are more commonly involved in Western populations where smoking and alcohol consumption are more prevalent. As stated previously, malignant transformation was most common in OLKs affecting the tongue (24.2%), followed by the tongue/ﬂoor of the mouth combined (14.9%), while the buccal mucosa had the lowest rate of malignant transformation (3.5%).

The oral microbiome also shows site specificity (11–13). Prior to the advent of next-generation sequencing (NGS), Mager et al. (12) used checkerboard DNA-DNA hybridisation to investigate the microbiomes of saliva, dorsum, ventral and lateral tongue, maxillary gingiva, floor of mouth, buccal mucosa, labial mucosa, and hard palate in 225 individuals. Saliva and lateral and dorsal tongue samples clustered together, with the other sites forming a second cluster. There were significantly higher proportions of Gram-negative, and mostly anaerobic, organisms such as *Veillonella parvula, Prevotella melaninogenica, Eikenella corrodens, Neisseria mucosa, Actinomyces odontolyticus, Fusobacterium periodonticum, Fusobacterium nucleatum* subsp. *vincentii*, and *Porphyromonas gingivalis* in the saliva/dorsal/lateral tongue cluster, while the second cluster was dominated by Gram-positive aerobes *Streptococcus mitis* and *Streptococcus oralis* and a single Gram-negative anaerobe, *Selenomonas noxia*. Aas et al*.*'s (11) findings, pre-NGS using 16S rRNA cloning, were broadly similar, although the dominant colonisers of the tongue differed, likely because of the different methodologies used for bacterial identification.

Finally, Segata et al. (13) sampled 200 subjects at seven oral sites: buccal mucosa, attached gingiva, hard palate, saliva, tongue, and two tooth surfaces along with tonsils, throat, and stool. Clustering similar to that in Mager et al*.*'s study was identified with buccal mucosa, gingiva, and hard palate clustered together (Group 1), while saliva and tongue grouped with throat and tonsils (Group 2). The Group 1 cluster consisted mostly of Firmicutes, followed by Proteobacteria, Bacteroidetes, Actinobacteria, and Fusobacteria, while the Group 2 cluster had a reduced relative abundance of Firmicutes and increased abundance of Bacteroidetes, Fusobacteria, Actinobacteria, and TM7. At the genera level, Group 1 was dominated by *Streptococcus*, while Group 2 had a more even distribution of *Streptococcus, Veillonella, Prevotella, Neisseria, Fusobacterium, Actinomyces*, and *Leptotrichia*.

**Figure 1:**
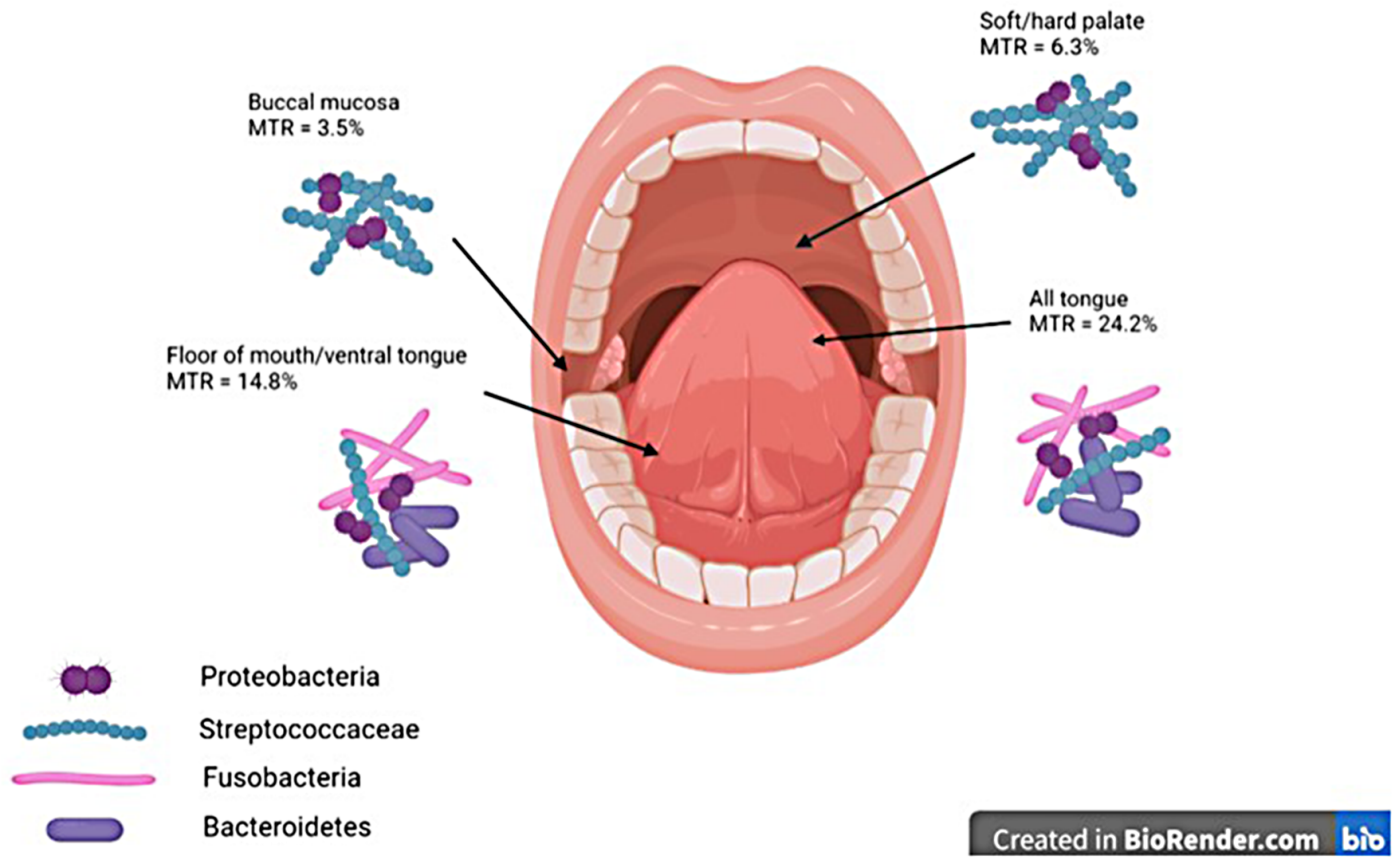
Graphic demonstrating MTRs at different oral mucosal sites and dominant taxonomic groups of bacteria at each site. MTRs, malignant transformation rates.

It is thus clear that the tongue, a site known to have high rates of malignant transformation, also has high levels of colonisation with Gram-negative organisms of phyla Fusobacteria and Bacteroidetes, whereas the buccal mucosa and palate, which have much lower rates of malignant transformation, are predominantly colonised by Gram-positive *Streptococci* (Figure 1).

## 5. Influence of smoking, betel nut, and electronic cigarettes on the oral microbiome

Along with site, smoking is also known to have a profound effect on the oral microbiome, although as for OSCC microbiome studies, results differ because of variations in sampling methodology and failure to control for factors such as periodontal disease for which smoking is an established risk factor. Depletion of *Neisseria* spp*.* in smokers is widely reported and many of these studies also report an increase in Fusobacteria (Table 1) (10, 14–17, 46–48). A switch to a community rich in Fusobacteria may increase the inflammatory stimulation on mucosal surfaces; *Fusobacterium nucleatum*, in particular, has been shown to induce IL-6-mediated inflammation (49). However, smoking is also a risk factor for periodontal disease, which, in turn, can increase the burden of Gram-negative bacteria in the oral cavity and may explain why some studies identify increases in *Fusobacterium nucleatum* in smokers. What influence, if any, this has on OSCC risk or malignant transformation of OLK has yet to be elucidated.

**Table 1:** Significant differences between the oral microbiomes of smokers and non-smokers in the literature.

Author	Sampling method and cohort	Periodontal health	Increased in smokers	Decreased in smokers
Thomas et al. (2014)	Mucosal swabs 13 smokers 9 never smokers	Not measured	*Fusobacteria* *Actinobacteria* *Capnocytophaga* *Prevotella*	Firmicutes *Cyanobacteria* TM7 BD1-5 *Neisseria* *Peptostreptococcus* *Gemella* *Granulicatella* *Staphylococcus*
Moon et al. (2015)	Subgingival plaque 134 smokers 134 non-smokers	Moderate periodontitis	Fusobacteria *Fusobacterium Streptococcus Veillonella Corynebacterium TM7* *Filifactor* *Parvimonas* *Tanerella* *Rothia*	Bacteroidetes *Neisseria* *Prevotella* *Aggregatibacter* *Selenomonas* *Veillonellaceae GQ422718*
Mason et al. (2015)	Subgingival plaque 200 subjects	Periodontally healthy	*Fusobacterium nucleatum* *Fusobacterium naviforme* *Filifactor alocis* *Dialister microaerophile Megasphaera sueciensis* *Megasphaera geminatus* *Megasphaera elsdenii* *Megasphaera micronuciformis Acinetobacter johnsonii* *Acinetobacter guillouiae* *Acinetobacter schindleri* *Acinetobacter baumannii* *Acinetobacter haemolyticus Pseudomonas pseudoalcaligenes* *Pseudoramibacter alactolyticus*	*Streptococcus sanguinis* *Streptococcus parasanguinis* *Streptococcus oralis* *Gemella elegans* *Gemella adiacens* *Actinomyces viscosus* *Actinomyces israelii* *Actinomyces dentalis* *Neisseria subflava* *Haemophilus parainfluenzae*
Wu et al. (2016)	Saliva 112 smokers 571 former smokers 521 never smokers	Not measured, no oral examinations	Actinobacteria Firmicutes *Atopobium Streptococcus* *Bifidobacterium Lactobacillus* *Veillonella*	Proteobacteria *Neisseria* *Haemophilus* *Aggregatibacter* *Capnocytophaga* *Corynebacterium* *Porphyromonas* *Prevotella* *Peptostreptococcus Leptotrichia* *Abiotrophia* *Selenomonas*
Amer et al. (2017)	Mucosal swabs (OLK study, subset analysis) 19 current smokers 10 former smokers 7 never smokers	Not measured		*Fusobacterium nucleatum* *Neisseria* *Leptotrichia*
Hsiao et al. (2018)	Saliva 104 male smokers 47 male never smokers	Not measured	*Fusobacterium nucleatum*	
Wirth et al. (2020)	Saliva 11 smokers 11 non-smokers (8 never, 3 former—>1 year cessation)	Dental examinations, moderation and severe periodontitis excluded	*Prevotella* *Megasphaera*	*Neisseria* *Capnocytophaga* *Porphyromonas* *Oribacterium*
Gopinath et al. (2021)	Buccal mucosa swabs 17 male smokers 13 male non-smokers	Not measured, no oral examinations	*Fusobacterium* spp. including *Fusobacterium nucleatum* *Saccharibacterium* spp. *Shuttleworthia* spp.	

OLK, oral leukoplakia.

To date, few studies have investigated the influence of betel nut or electronic cigarette (e-cigarettes) use on the oral microbiome. Hernandez et al. (50) found significantly increased abundances of *Streptococcus infantis* and reduced abundances of *Parascardovia* and other *Streptococci* in current betel nut chewers, while Hsiao et al. (16) associated betel nut use with an increased abundance of *Prevotella intermedia*. E-cigarette use has been associated with increased abundances of *Veillonella* and *Porphyromonas* (51–54).

## 6. Microbial methods of carcinogenesis

To date, only one study has shown that bacteria may induce malignant transformation in the oral mucosa, where *Porphyromonas gingivalis* and *Fusobacterium nucleatum* were shown to promote carcinogenesis in a chemically induced murine model of tongue OSCC (55). The exact mechanisms of microbial carcinogenesis are still unclear, although research suggests that bacterial-induced epithelial change, promotion of cellular invasion and proliferation, acetaldehyde (ACH) production, and inflammation are likely involved.

The epithelial adhesion molecule E-cadherin, a marker of the epithelial state, is lost early in oral dysplasia (56) and is considered an early event in epithelial-mesenchymal transition (EMT), a biological process whereby an epithelial cell changes to a mesenchymal cell phenotype and is therefore able to migrate, invade, and resist apoptosis. EMT has, therefore, been associated with the initiation of carcinogenesis and facilitation of cancer spread. Bacteria can induce EMT, with *Helicobacter pylori* being a well-known example, inducing EMT *via* its cytotoxin-associated gene A (Cag-A) (57). Long-term infection with *Porphyromonas gingivalis* has also been associated with initial EMT changes in oral epithelial cells, including decreased E-cadherin expression and upregulation of Vimentin (58). Zhang et al. (49) recently showed that the *Fusobacterium nucleatum* adhesin, FadA, can facilitate EMT *via* downregulation and transfer of E-cadherin to the cytoplasm and upregulation of N-cadherin, Vimentin, and SNAI1 and miRNA MIR4435-2HG, thus providing a possible mechanism for the role of *Fusobacterium nucleatum* in carcinogenesis. *Fusobacterium nucleatum* is also known to promote cell proliferation *via* upregulation of multiple kinases (59), while both *Fusobacterium nucleatum* and *Porphyromonas gingivalis* promote cellular invasion (59, 60).

Microbial production of ACH is also likely to play a role in oral carcinogenesis. ACH, produced by the reduction of ethanol by host or microbial acetaldehyde dehydrogenase (61), is designated as a Class 1 carcinogen by the IARC (62, 63) and is a known risk factor for OSCC. ACH is a genotoxic carcinogen capable of inducing DNA damage and secondary hyperproliferation of epithelium (61, 64–67). Mutagenic levels (>100 µM) of ACH can be detected in saliva following the ingestion of 0.5 g alcohol per kg body weight. Smoking also significantly increases salivary ACH production, with active smoking resulting in an additional 200–400 µM peak ACH concentration in saliva for the duration of the smoking (64, 68–70). Interestingly, the amount of ACH produced is significantly reduced after 3 days of using a chlorhexidine mouthrinse (68), supporting the role of bacteria in ACH production.

All species of *Neisseria*, along with *Rothia mucilaginosa, Streptococcus mitis*, and *Prevotella histicola*, have been shown to produce ACH from ethanol (71–73). As *Neisseria* are known to be decreased in smokers (72), it is likely that other bacteria are responsible for ACH production in this cohort. *Rothia mucilaginosa* strains isolated from dysplastic OLKs have been shown to be capable of producing mutagenic levels of ACH in the presence of alcohol (74), while other studies have shown that certain *Streptococci,* particularly viridans group *Streptococci*, have significant ACH-producing ability. However, *Streptococci* are typically associated with a healthy microbiome and reduced in OPMDs and OSCC (16, 73, 75). Therefore, it is clear that more studies are needed to investigate the role of bacteria in ACH production and the various factors that influence its production; however, it is apparent that many bacteria within the normal oral microbiome are potent producers of ACH, raising questions about their role in oral carcinogenesis.

Finally, inflammation, which has been recognised as a fundamental component of malignant processes (76), is another potential link between the oral microbiome and OSCC. Chronic infection causes inflammation, and as demonstrated by the associations between hepatitis and hepatocellular carcinoma (HCC) and *Helicobacter pylori–*induced gastritis and gastric cancer, this infection-induced inflammation can precede cancer development (76). The role of infection-associated inflammation in OSCC is more complex as the oral cavity harbours hundreds of bacteria and fungi as commensals without being in a constant state of inflammation. However, polymicrobial dysbiosis in the oral cavity does lead to inflammation, as demonstrated by periodontal disease, and therefore may also play a role in carcinogenesis. Bacterial pathogens can stimulate host immune cells, such as macrophages and neutrophils, and oral epithelial cells, *via* their surface receptors to activate the STAT3 and NF-*κ*B signalling pathways, which, through the promotion of proliferation, angiogenesis, invasion, and enhanced survival, can play a role in tumorigenesis (77, 78). *Fusobacterium nucleatum*, in particular, has been shown to bind and enter host epithelial and endothelial cells and induce NK-*κ*B- and IL-6-mediated inflammation *via* its FadA adhesin molecule (79, 80).

Although the definitive pathways of bacterial carcinogenesis have yet to be elucidated, it is likely that, rather than a single bacteria or pathway, there is a polymicrobial dysbiosis and therefore multiple mechanisms are involved. This dysbiosis is likely to be influenced by smoking habit and it is also possible that due to baseline differences in mucosal site colonisation, some mucosal sites are more susceptible to colonisation by a potentially carcinogenic microbiome.

## 7. Discussion

OLK, the most common OPMD, is most commonly found on the buccal mucosa and tongue. However, OLKs at these sites display very different malignant transformation rates. In health, both sites have distinctly different microbial communities, which raises the question of the role of the microbiome in the malignant transformation of OLKs at these sites. While the microbiome may not initiate dysplastic change, sites with a significantly higher burden of Gram-negative bacteria (e.g., tongue) may be subject to greater pro-inflammatory stimuli, which could play a role in driving abnormal cellular phenotypes. OLKs on the palate and buccal mucosa where *Streptococci* predominate may not be exposed to the same level of inflammatory stimulation.

Smoking, a major risk factor for oral cancer, also significantly influences the oral microbiome, depleting common oral taxa such as *Neisseria* spp., which may allow pathobionts to proliferate. Smoking may also contribute to the Gram-negative burden through its role in promoting periodontal disease. It is not yet known whether this effect of smoking on the microbiome contributes to oral carcinogenesis. However, it is clear that smoking is a potential confounding factor in establishing the carcinogenic potential of the oral microbiome. In future, site-specific studies, with anatomically matched control sites, in non-smokers and smokers may provide answers to these questions.
